# A Survey of the Healthcare Workers in Afghanistan during the COVID-19 Pandemic

**DOI:** 10.4269/ajtmh.20-1367

**Published:** 2020-12-11

**Authors:** Arash Nemat, Abdullah Asady, Nahid Raufi, Naqeebullah Zaki, Ehsanullah Ehsan, Noor Ahmad Shah Noor, Qingchun Zeng

**Affiliations:** 1 Department of Microbiology, Kabul University of Medical Sciences, Kabul, Afghanistan;; 2 Department of Cardiology, Nanfang Hospital, Southern Medical University, Guangzhou, China;; 3 Department of Dermatology, Kabul University of Medical Sciences, Kabul, Afghanistan;; 4 Department of Dermatology, Guangdong Provincial Dermatology Hospital, Southern Medical University, Guangzhou, China;; 5 Department of Public Health, Tata Institute of Social Sciences, Mumbai, India;; 6 Department of Dermatology, Alberoni University, Kapisa, Afghanistan;; 7 Department of Oral and Maxillofacial Surgery, Kabul University of Medical Sciences, Kabul, Afghanistan

## Abstract

Healthcare workers (HCWs) in Afghanistan faced many challenges during the COVID-19 pandemic. A cross-sectional, online survey was conducted from July 4, 2020 to July 12, 2020 to evaluate the working conditions and health situation of HCWs in Afghanistan during the pandemic. Healthcare workers from 34 provinces, who were actively working in Afghan hospitals, were invited to participate in this study; 925 HCWs completed the survey. The results showed that 85% of the HCWs participated in the study were tested positive for COVID-19. This highlights the critical need of HCWs for personal protective equipment when caring for suspected and/or confirmed cases of the COVID-19.

## INTRODUCTION

COVID-19 emerged from Wuhan, China, in December 2019 and has spread to almost all countries throughout the globe, with numerous new cases/deaths being reported every day.^1^ Scientific evidence shows that COVID-19 is being transmitted between people in close contact by air droplets.^2^ Globally, as of July 12, 2020, there were 12,401,262 confirmed cases of COVID-19, including 559,047 deaths reported by the WHO.^3^

In Afghanistan, the first positive case of COVID-19 was reported from Herat Province on February 24, 2020. It was a 35-year-old man who had recently visited Qom, Iran.^4^ A few days later, the highly contagious virus was found to have spread to several other locations within the country. As of July 2020, a total of 35,526 cases and 1,185 deaths due to COVID-19 have been confirmed in Afghanistan.^5^ It is said that the actual figures of the infected cases and deaths could be higher than what is reported.^6^

Healthcare workers (HCWs), due to the inherent nature of their profession, are at particularly high risk of catching COVID-19 infection. As of July 2020, the United Nations announced that worldwide more than 1.4 million infections of COVID-19 were accounted for in HCWs; this was 10% of all cases.^7^

Afghanistan has an estimated population of 31.6 million. Among them are about 9.4 skilled HCWs and 1.9 physicians, per 10,000 inhabitants. They are unevenly distributed across the country and face many challenges.^8^ Because of inadequate government support and lacking personal protective equipment (PPE), HCWs in Afghanistan got infected with COVID-19 and lost their lives in disproportionately large numbers.^9^ The cross-sectional survey presented here aimed at evaluating the working conditions and health situation of HCWs in Afghanistan during the COVID-19 pandemic in the month of July 2020.

## MATERIALS AND METHODS

This was a cross-sectional online survey conducted among HCWs in Afghanistan. A 10-item questionnaire was developed and distributed using SurveyMonkey. Participants were recruited through social media (Facebook and WhatsApp) from an existing association of Afghan HCWs. The questionnaire was delivered in English as the targeted study participants could understand English. Those who volunteered to answer the questionnaire were allowed to complete it only once and were given the opportunity to terminate it at any time within the study period. The survey was anonymous, and the data provided by the participants were kept confidential. An introductory paragraph described the aim of the study, and an informed consent was obtained from the participants. The survey was prepared by the members of the Department of Microbiology of Kabul University of Medical Sciences. The questionnaire was developed to assess the participants’ behavior regarding personal safety and their concern during the COVID-19 pandemic.

Three items of the questionnaire addressed demographic information. This included, gender (male and female), age in categories (18–24, 25–34, 35–44, 45–54, 55, or > 55 years) and occupation (medical specialist, medical trainee, dentist, nurse, midwife, anesthesiologist, laboratory technician, physiotherapist, administration staff, and other healthcare professionals). The questionnaire enquired about monthly salary of HCWs from less than 10,000 AFG/130 USD up to more than 50,000 AFG/650 USD. Other questions addressed access to PPE (disposable gloves, gowns, masks, N95 masks, facial protective shields, and safety glasses) and asked whether the respondents/HCWs provided for personal safety through their own finances, whether medical staff had sufficient knowledge about COVID-19, whether they were tested positive for COVID-19, and what clinical symptoms (fever, cough, respiratory distress, sore throat, runny nose, loss of sense of smell and taste, fatigue, headache, muscle pain, ocular pain, and others) they experienced. The questionnaire also asked about their satisfaction with their local health authorities and about other main concerns they had. Descriptive statistics, frequencies, and percentages were used to summarize the data.

## RESULTS

A total of 925 HCWs completed the online survey. Of them, 299 (32%) were medical specialists, 341 (37%) medical trainees, 75 (8%) dentists, 64 (7%) laboratory technicians, 29 (3%) nurses, and the rest belonged to other healthcare disciplines. Most participants 619 (67%) were in the 25- to 34-year-old category (Table 1).

**Table 1:** Demographic characteristics of the healthcare workers

Characteristic	Category	*n* (%)
Gender	Female	114 (12%)
	Male	811 (88%)
Age (years)	18–24	87 (9%)
	25–34	619 (67%)
	35–44	145 (16%)
	45–54	37 (4%)
	55 Or more	37 (4%)
Medical profession	Medical specialist	299 (32%)
	Medical doctor/trainee	341 (37%)
	Dentist	75 (8%)
	Nurse	29 (3%)
	Midwife	17 (2%)
	Anesthesiologist	28 (3%)
	Laboratory technician	64 (7%)
	Physiotherapist	10 (1%)
	Administration worker	44 (5%)
	Others	18 (2%)

Participants indicated that they had access to the following essential items: one-layer medical masks produced inside Afghanistan 378 (41%), medical masks 451 (49%), N95 masks 206 (22%), disposable gloves 574 (62%), goggles 186 (20%), disposable gowns 215 (23%), and face shields 228 (25%) (Table 2). Among them, 632 (68%) study participants answered that they always provided PPE through their own finances, 162 (18%) sometimes provided PPE through their own finances, and only 131 (14%) had access to PPE for free. In terms of monthly salary, 206 (22%) of respondents earned less than 10,000 AFG/130 USD and 240 (26%) earned 10,000–20,000 AFG/130–260 USD (Table 3).

**Table 2:** Essential protective item access for Afghan healthcare workers

Personal protective equipment	*n* (%)
Medical masks produced inside Afghanistan	378 (41%)
Medical masks	451 (49%)
N95 masks	206 (22%)
Disposable gloves	574 (62%)
Goggles	186 (20%)
Disposable gowns	215 (23%)
Face shields	228 (25%)

**Table 3:** Monthly salary of Afghan healthcare workers

AFG	Equivalent USD	*n* (%)
Less than 10,000	Less than 130	206 (22%)
10,000–20,000	130–260	240 (26%)
20,000–30,000	260–390	112 (13%)
30,000–40,000	390–520	198 (21%)
40,000–50,000	520–650	54 (6%)
More than 50,000	More than 650	115 (12%)

The majority of the study participants 814 (88%) reported that they had sufficient knowledge about COVID-19. When asked about their level of satisfaction with the services offered by public health authorities, particularly in providing PPE, 54 (6%) of participants were very satisfied, 183 (20%) satisfied, 262 (28%) neither satisfied nor dissatisfied, 258 (28%) dissatisfied, and 168 (18%) were very dissatisfied.

Regarding their main concerns during the COVID-19 pandemic, the following issues were reported by the study participants: spreading the virus to family members 577 (62%), lack of PPE 426 (46%), acquiring the virus from their colleagues and patients 401 (43%), lack of necessary equipment/medicine 352 (38%), work overload 302 (33%), absence of a suitable place for accommodation 282 (30%), and delays in salary payments 289 (31%). In response to the question “Have you been tested positive for COVID-19?” 788 (85%) participants answered “yes,” 66 (7%) were “not sure,” and only 71 (8%) answered “no.” In response to the question regarding COVID-19 symptoms, the most common symptom among the study participants who tested positive for COVID-19 was muscle pain 574 (73%), followed by fever 548 (70%), sore throat 506 (64%), headache 488 (62%), fatigue 476 (60%), and cough 417 (53%) (Table 4).

**Table 4:** Clinical symptoms of healthcare workers infected with COVID-19 in Afghanistan

Symptom	*n* (%)
Muscle pain	574 (73%)
Fever	548 (70%)
Sore throat	506 (64%)
Headache	488 (62%)
Fatigue	476 (60%)
Cough	417 (53%)
Runny nose	259 (33%)
Anosmia	248 (31%)
Shortness of breath	143 (18%)
Ocular pain	131 (17%)

## DISCUSSION

To the best of our knowledge, this is the first cross-sectional survey conducted among HCWs in Afghanistan. In this online survey, 925 HCWs participated from across the country. Most respondents were medical trainees and specialists actively working in Afghan hospitals. This study indicates that more than half of the participants (68%) provided basic PPE using their own money, whereas only 14% received PPE from relevant government bodies. This could highlight the critical lack of PPE for the frontliners in the fight against COVID-19. The perception of the HCWs about limited support and supplies from public health authorities and medical institutions in regard to their personal safety suggests that improvement is needed in these areas. Moreover, 82% of the HCWs expressed that they earned less than 40,000 AFG/520 USD salary/month for a full-time employment in a government hospital. Even with such limited salary, Afghan HCWs had to purchase their PPE from their personal budget. In addition, almost one-third (31%) of the HCWs declared that their monthly salaries were not paid on time. This could further aggravate the situation, particularly in a place where most HCWs should pay for their PPE.

**Figure 1.**
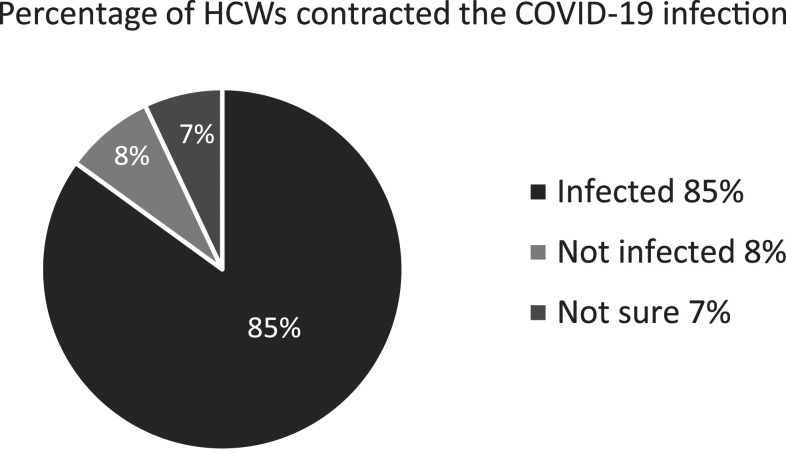
Percentage of healthcare workers (HCWs) who contracted the COVID-19 infection.

A notable finding of the study was that more than three-quarters (85%) of the HCWs were tested positive for COVID-19. This shows an alarmingly high rate of the infection among HCWs (Figure 1) . This could possibly be because of scarcity of PPE in hospital environments for HCWs and massive exposure of people to COVID-19. This study highlights the reality and perceptions about the safety and resources available for HCWs during the COVID-19 pandemic in Afghanistan. It also informs the authorities about the urgent need for attention to the alarmingly high rate of infection and scarcity of PPE among HCWs in Afghanistan.

### Limitations of the study.

Many HCWs in Afghanistan, particularly those who live in remote areas, could not participate in this survey because of lack of access to the Internet. Participation in this study is biased because only those HCWs who had access to social media, particularly Facebook and WhatsApp, could participate. This study had limited scope. Participants were asked to answer very specific questions that might not cover all issues involved in the complex situation of the personal safety of HCWs. Regarding statistics, no power calculations were undertaken before the beginning of this survey. However, by definition, its purpose was descriptive only. Moreover, because of the limited number of COVID-19 test centers in Afghanistan, it was difficult to assess the accuracy of test results and infection rates.

## CONCLUSION

The protection and support of HCWs are supposed to be public health priorities, particularly during a pandemic. In this survey, we report an alarmingly high rate of COVID-19 infection among HCWs and highly limited access to essential PPE during the COVID-19 pandemic in Afghanistan. It highlights that any possible resources should be made available for them urgently to protect themselves, stay healthy, and able to continue caring for patients.
